# Structure of giant muscle proteins

**DOI:** 10.3389/fphys.2013.00368

**Published:** 2013-12-12

**Authors:** Logan C. Meyer, Nathan T. Wright

**Affiliations:** Department of Chemistry and Biochemistry, James Madison UniversityHarrisonburg, VA, USA

**Keywords:** titin, nebulin, obscurin, immunoglobulin, fibronectin type III, structure

## Abstract

Giant muscle proteins (e.g., titin, nebulin, and obscurin) play a seminal role in muscle elasticity, stretch response, and sarcomeric organization. Each giant protein consists of multiple tandem structural domains, usually arranged in a modular fashion spanning 500 kDa to 4 MDa. Although many of the domains are similar in structure, subtle differences create a unique function of each domain. Recent high and low resolution structural and dynamic studies now suggest more nuanced overall protein structures than previously realized. These findings show that atomic structure, interactions between tandem domains, and intrasarcomeric environment all influence the shape, motion, and therefore function of giant proteins. In this article we will review the current understanding of titin, obscurin, and nebulin structure, from the atomic level through the molecular level.

Titin, obscurin, and nebulin are all giant muscle-specific proteins that play key roles in sarcomere organization, strength, and development. The size (all >500 kDa) and apparent flexibility of these molecules has hindered traditional structure determination. However, through the integration and creative use of multiple structural elucidation tools including crystallography, nuclear magnetic resonance (NMR), small angle x-ray scattering (SAXS), and electron microscopy (EM), a concerted effort to describe fully the structure and dynamics of these systems is underway. Here, we review the current understanding of giant muscle protein structure. Titin and obscurin are primarily composed of related but distinct modular domains, while nebulin has a more repetitive and simple repeating structure.

## Ig and FnIII domains

**Figure 1:**
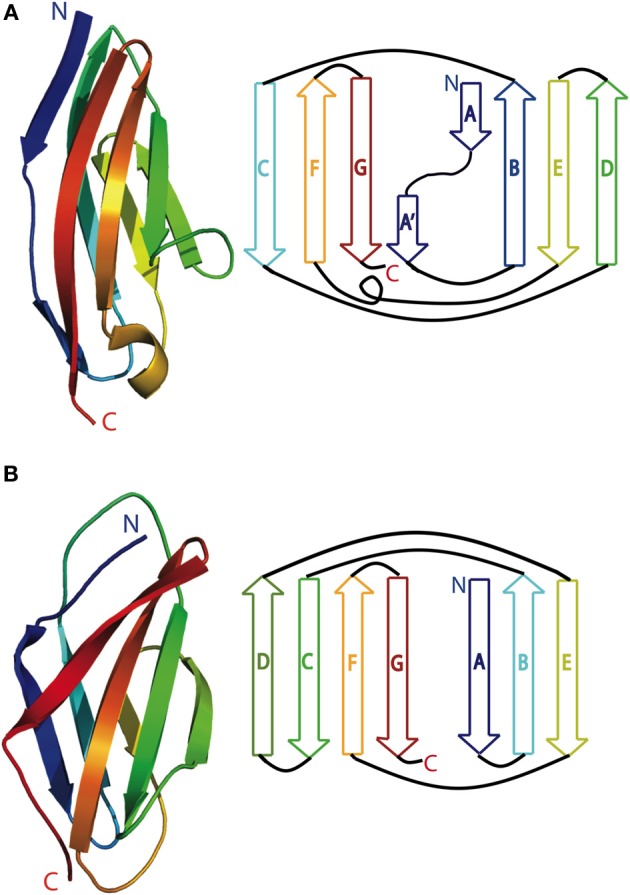
**Cartoon (left) and schematic (right) of a typical (A)** Immunoglobulin-like domain (M7 of titin; pdb 3PUC) and **(B)** fibronectin type III domain (titin domain A77; pdb 3LPW). Blue is the N-terminus and red is the C-terminus.

Both titin and obscurin are made up predominantly of either Ig (Immunoglobulin) or FnIII (Fibronectin type III)-like domains. Titin can have close to 300 domains and obscurin can contain almost 70, depending on the isoform [reviewed in Kontrogianni-Konstantopoulos et al. (2009)]. Each of these domains is around 100 residues in length, and folds independently (Pfuhl and Pastore, 1995; Pfuhl et al., 1995; Improta et al., 1996). Both kinds of domains are comprised of two β sheets packed face-to-face to form a β-sandwich held together by a conserved hydrophobic core (Campbell and Spitzfaden, 1994; Harpaz and Chothia, 1994). The FnIII-like fold is comprised of antiparallel β-strands ABE forming the first sheet and DCFG in the second sheet (Figure 1B). Ig folds are similar, except that the D strand is located in the first β-sheet (Figure 1A) and an additional flanking β-strand on the second sheet can also be incorporated into the Ig fold. Hence strands ABED form the first sheet and strands (Cʹ)CFG(Aʹ) create the second. Most Ig folds of both titin and obscurin belong to the intermediate I-set type of the Ig superfamily [so named due to their shared, or intermediate, characteristics between C (constant) and V (variable) domains in antibodies] (Harpaz and Chothia, 1994; Pfuhl and Pastore, 1995). Giant muscle protein Ig-like folds can deviate from the prototypical Ig fold through inclusion of noncanonical A, Aʹ, and Cʹ strand structure, as well as having fewer interactions between the Aʹ-B and E-F loops (Tskhovrebova and Trinick, 2004).

In both titin and obscurin, the Ig domains have ~40% sequence conservation (Witt et al., 1998; Fraternali and Pastore, 1999; Young et al., 2001). Most of the highly conserved residues are located in the core of the β-sandwich. This results in a pairwise backbone RMSD of <1.5 Å between Ig domains. In contrast, the solvent-exposed residues differ substantially between the domains, and are the basis of titin and obscurin domains' target binding specificity (Mueller et al., 2007; Kontrogianni-Konstantopoulos et al., 2009; Pernigo et al., 2010). Recent papers have shown that the orientation between tandem domains also can be important for normal protein function (Pinotsis et al., 2006; Zou et al., 2006).

### Ig and FnIII structure determination

Individual FnIII and Ig domains fold independently, can be easily purified, and are stable in high concentrations at room temperature for months. High-resolution structures of titin and obscurin Ig and FnIII domains show that both crystallography and NMR methods result in reliably accurate structures (see Table 1). Complementing these methods, SAXS experimentation is used to generate most low-resolution structures in the literature, although cryo-EM is also sometimes employed [for instance: (Von Castelmur et al., 2008; Jeffries et al., 2011; Al-Khayat et al., 2013) for cryo-EM and (Marino et al., 2005; Vazina et al., 2006; Von Castelmur et al., 2008; Bucher et al., 2010; Tskhovrebova et al., 2010) for SAXS]. For X-ray crystallography, titin, and obscurin domains most frequently crystallize in various concentrations of ammonium sulfate. Resolutions for single Ig domains vary between 0.96 and 2.10 Å, and resolution of multiple tandem domains is between 1.40 and 3.30 Å. NMR structures are conducted from 25 to 37°C, at pH values 4.8–7.5 in medium salt (usually 100 mM NaCl, 20 mM Tris-*d11*) and with protein concentrations between 5 and 11 mg/mL, using traditional heteronuclear multidimensional NMR techniques. SAXS experiments, often conducted on tandem-domain constructs, are usually done at pH 7.5 values, 15–20°C using a wide range of salt (50 mM–1 M NaCl). Protein concentrations between 1 and 25 mg/mL are used for these trials. All SAXS experiments are run at multiple concentrations, with no reported systematic errors resulting from protein aggregation.

**Table 1:** **Domain structures of titin, obscurin, and nebulin**.

**Domain**	**Type**	**Location**	**PDB**	**Res. (Å)**	**Method**	**References**
**TITIN**						
**Z-disc**						
Z1Z2—Telethonin	Ig-repeat	Z-disc	2F8V	2.75	X-Ray	Pinotsis et al., 2006
Z1Z2—Telethonin	Ig-repeat	Z-disc	1YA5	2.44	X-Ray	Zou et al., 2006
Z1Z2	Ig-repeat	Z-disc	2A38	2.00	X-Ray	Marino et al., 2006
Z7 repeat-α-actinin	Z-repeat	Z-disc	1H8B		NMR	Atkinson et al., 2001
**I-band**						
I1	Ig-like	I-band	1G1C	2.10	X-Ray	Mayans et al., 2001
I27	Ig-Like	I-band	1WAA	1.80	X-Ray	Stacklies et al., 2009
I27	Ig-Like	I-band	1TIT		NMR	Improta et al., 1996
I65-I70	Ig-repeat	I-band	3B43	3.30	X-Ray	Von Castelmur et al., 2008
I67-I69	Ig-repeat	I-band	2RIK	1.60	X-Ray	Von Castelmur et al., 2008
**A-band**						
A71	FnIII	A-band	1BPV		NMR	Goll et al., 1998
A77-A78	FnIII Tandem	A-band	3LPW	1.65	X-Ray	Bucher et al., 2010
A164-A165	Ig-repeat	A-band	3LCY	2.50	X-Ray	Steward et al., 2012
A168-A169	Ig-repeat	A-band	2J8O	2.50	X-Ray	Mueller et al., 2007
A168-A169	Ig-repeat	A-band	2J8H	1.99	X-Ray	Mueller et al., 2007
A168-A170	Ig-repeat	A-band	2NZI	2.90	X-Ray	Mrosek et al., 2007
**M-line**						
Serine kinase	Serine Kinase	M-line	1TKI	2.00	X-Ray	Mayans et al., 1998
M1	Ig-Like	M-line	2BK8	1.69	X-Ray	Mueller et al., TBP
M4	Ig-Like	M-line	3QP3	2.00	X-Ray	Sauer et al., TBP
M5, N-term. extension	Ig-Like	M-line	1NCT		NMR	Pfuhl et al., 1997
M5	Ig-Like	M-line	1TNN		NMR	Pfuhl et al., 1995
M5	Ig-Like	M-line	1TNM		NMR	Pfuhl et al., 1995
M7	Ig-Like	M-line	3PUC	0.96	X-Ray	Sauer et al., TBP
M10-OL1	Ig-Like	M-line	3KNB	1.40	X-Ray	Sauer et al., 2010
M10 F17R—OL1	Ig-Like	M-line	2WWK	1.70	X-Ray	Pernigo et al., 2010
M10-OL1	Ig-Like	M-line	2WP3	1.48	X-Ray	Pernigo et al., 2010
M10-OL1 in P1	Ig-Like	M-line	2WWM	2.30	X-Ray	Pernigo et al., 2010
M10	Ig-Like	M-line	3Q50	2.05	X-Ray	Sauer et al., 2010
M10	Ig-Like	M-line	2Y9R	1.90	X-Ray	Pernigo et al., 2010
**OBSCURIN**						
Ig28 (KIAA1556)	Ig-Like		2EDF		NMR	RIKEN 2007
Ig29 (KIAA1556)	Ig-Like		2DKU		NMR	RIKEN 2007
Ig30	Ig-Like		2CR6		NMR	RIKEN 2005
Ig32 (KIAA1665)	Ig-like		2E7B		NMR	RIKEN 2007
Ig32 (KIAA1665)	Ig-Like		2YZ8	2.00	X-Ray	RIKEN 2007
Ig34	Ig-Like		2EDR		NMR	RIKEN 2007
Ig35	Ig-Like		2EDT		NMR	RIKEN 2007
Ig36	Ig-Like		2EDW		NMR	RIKEN 2007
Ig37	Ig-Like		2EDH		NMR	RIKEN 2007
Ig38	Ig-Like		2EDQ		NMR	RIKEN 2007
Ig38 (KIAA1556)	Ig-Like		2GQH		NMR	RIKEN 2006
Ig39	Ig-Like		2EDL		NMR	RIKEN 2007
Ig39 (KIAA1556)	Ig-Like		2DM7		NMR	RIKEN 2006
SH3	Serine kinase		1V1C		NMR	Pfuhl et al., TBP
PH	PH domain		1FHO		NMR	Blomberg et al., 2000
**NEBULIN**						
SH3	Serine kinase	Z-disc	1ARK		NMR	Politou et al., 1998

Obscurin fragments labeled KIAA1556 come from an alternatively spliced forms of obscurin. RIKEN is a structural genomics initiative; these pdbs are not associated with any manuscript citation. TBP denotes To Be Published. OL1 denotes the N-terminal Ig domain of obscurin-like 1.

### Ig and FnIII stability and dynamics

**Figure 2:**
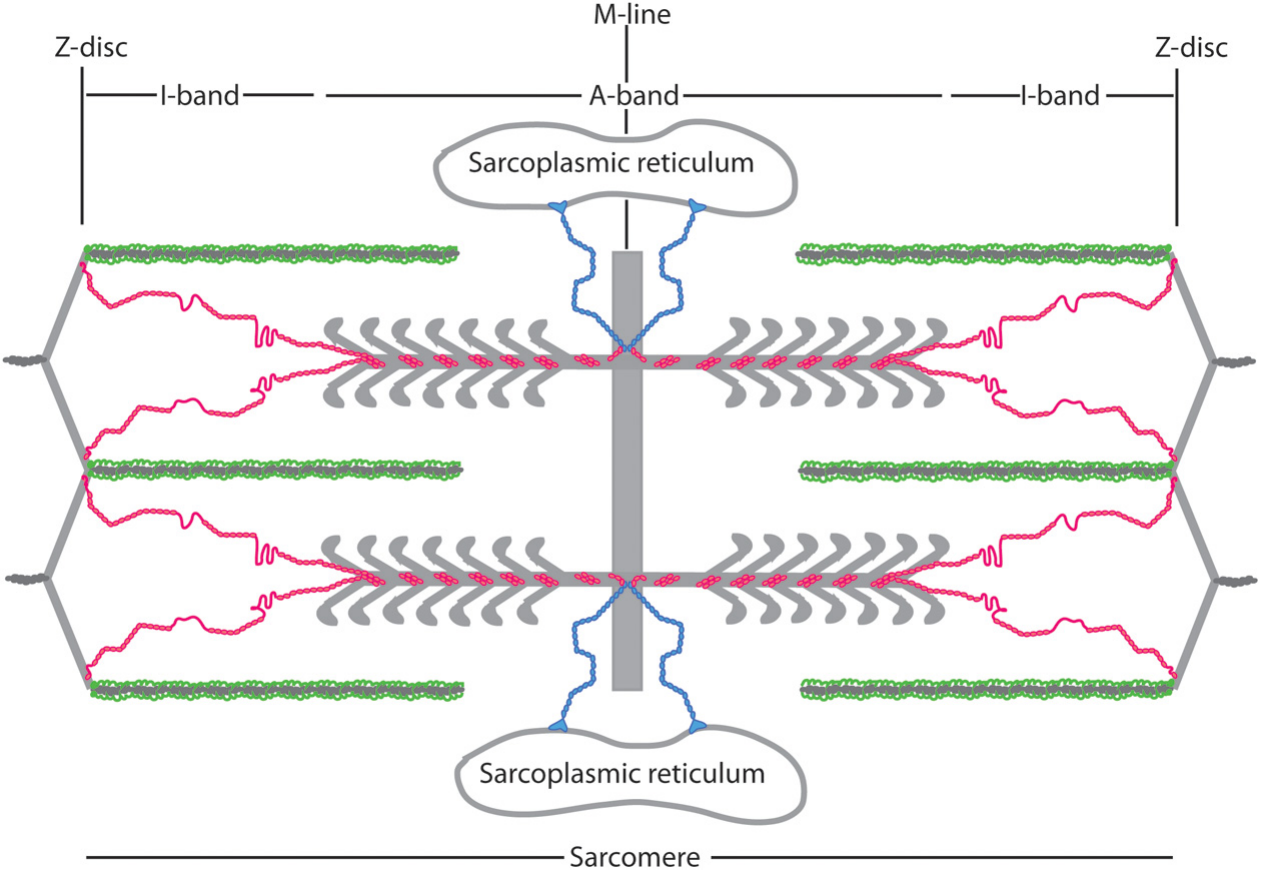
**Schematic of nebulin (green), obscurin (blue), and titin (red) arrangement in the sarcomere**. Nebulin is oriented with its C terminus in the Z-disc, Obscurin is oriented with its N-terminus in the M-line and its C-terminus bound to the sarcoplasmic reticulum, and titin is oriented with its N-terminus at the Z disc and its C-terminus at the M-line.

Due to its central role in muscle stability and stretch signaling, the mechanical and chemical stability of titin has been studied extensively. Individually expressed titin domains thermally unfold between 35 and 70°C, although domains from the I-band region of titin (see Figure 2 for reference) are typically more thermally and chemically stable than those from the A-band (Politou et al., 1995). Conditions that mimic molecular crowding environments generally increase stability (Bolis et al., 2004). NMR dynamics studies on representative Ig domains show that virtually the entire Ig domain, minus the N and C termini and some residues in loops, has order parameters of greater than 0.8, indicating that individual domains are extremely stable (Pfuhl et al., 1997; Nicastro et al., 2004).

Multiple papers describe the mechanical unfolding of titin domains [among others: (Rief et al., 1998, 2000; Li and Fernandez, 2003; Garcia et al., 2009; Stacklies et al., 2009; Yagawa et al., 2010)]. FnIII-like structures unfold at lower forces than Ig domains (100–200 pN vs. 150–300 pN) (Rief et al., 1998). Since FnIII domains are only found in the A-band, where titin interacts extensively with the thick filament, these domains likely do not significantly contribute to titin's mechanical stability *in vivo* (Wang et al., 1992). In the I-band, those Ig domains that are most easily unfolded (150 pN) are located near the Z-disc, while those with intermediate mechanical stability (~200 pN) are in the center of the I-band and those able to withstand the strongest forces (250–300 pN) are near the A/I junction (Rief et al., 1998; Li et al., 2002; Watanabe et al., 2002; Li and Fernandez, 2003). While the chance of domain unfolding in an intact sarcomere is low, such an event may occur if a muscle is severely overstretched, and unfolding would propagate from the Z-disc through the I-band toward the A/I junction. Ig domains can refold on the order of seconds after the tension on the polypeptide has abated and the protein molecule is fully relaxed (Kellermayer et al., 1997; Linke et al., 1998; Rief et al., 1998). One current model of titin's behavior in stretch-response is that the mechanical unfolding/refolding phenomenon protects the protein from irreversible damage due to severe muscle overstretching [For more a more thorough review on this topic, please see (Tskhovrebova and Trinick, 2004)]. Of the 37 Ig-like domains of giant muscle proteins that have been solved (see Table 1), only one (titin I1) contains a disulfide bond (Mayans et al., 2001). This bond is between residues in the C and E strand, instead of the more typical B and F strand connection. The presence of disulfide bonds within titin Ig domains, especially in the I-band, may confer added stability to mechanical stretch and be involved in the cellular reaction oxidative stress caused by muscle injury or fatigue (Mayans et al., 2001).

## Titin architecture within the sarcomere

Titin is the largest known polypeptide, and spans 1 μm across half of the sarcomere (see Figure 2 for reference). Its N-terminus is anchored in the Z-disc and interacts extensively with other Z-disc proteins (Gautel et al., 1999). In the neighboring I-band region, titin is made up primarily of Ig domains, the presumably unstructured N2A and N2B region, and the PEVK region. The titin I-band region participates in fewer protein-protein interactions than the rest of the molecule and contributes to most of titin's elastic properties. The A-band region of titin is comprised primarily of FnIII-like and Ig domains, and has extensive interactions with myosin, Myosin Binding Protein C (MyBP-C), and other thick filament-associated proteins [reviewed in Kontrogianni-Konstantopoulos et al. (2009)]. The C-terminus of titin, discussed in the next paragraph, is embedded in the M-line and contains a kinase domain and Ig domains that are separated by unstructured M-insertions.

Direct mechanosensing is facilitated through the only enzymatically active region in titin, the M-line kinase domain. Recent papers have shown that this region is activated through some degree of mechanical unfolding (at forces >30 pN), which in turn signals the myocytes to recognize and adjust to the changing forces of muscle contraction (Linke, 2008; Puchner et al., 2008; Linke and Kruger, 2010; Gautel, 2011a). Loss of this mechanical sensory ability results in malformed sarcomeres and, more globally, in cardiac atrophy and death in mice (Peng et al., 2005; Weinert et al., 2006). Other parts of titin, while likely also important for mechanosensing, have no intrinsic enzymatic activity, and thus signal stretch activation must occur indirectly through protein-protein interactions.

### Titin structure at the Z-Disc

There are 10 Ig domains of titin embedded in the Z-disc of the sarcomere. For a general schematic of the entirety of the titin molecule in relationship to the sarcomere, please see (Kontrogianni-Konstantopoulos et al., 2009). High-resolution structures exist for two regions in the Z-disc of titin; the extreme N-terminus (Z1Z2, denoting the 1st and 2nd domain within the Z-disc of the sarcomere; this kind of nomenclature is the most common way of labeling various titin domains) and a small peptide between the 3rd and the 4th Ig domain. This peptide is part of the second Z-insertion (the first one is between Z2 and Z3). When bound to the cleft in the EF-hand of α-actinin (*K*_D_ from 100 nM to 4 μM), part of this Z-insertion forms an α helix (Joseph et al., 2001).

The tandem domains Z1 and Z2 were crystalized in two different orientations within the same crystal (Marino et al., 2006). In both of these conformations, the individual Ig domains are near-identical in structure and temperature factor; however, the relative orientation of Z1 to Z2 changes considerably from an overall “V” shape to being fully extended. SAXS and NMR data indicate that the Z1Z2 solution structure is relatively extended, but not to the degree that the X-ray data show (Marino et al., 2005, 2006). NMR HSQC data show that residues in the hinge region between Z1 and Z2 have equally strong signals compared to other residues in the protein, and these linker residues only show one peak per residue. Such data are consistent with a linker region that is not highly dynamic. When combined with the X-ray data, it becomes apparent that while the Z1Z2 molecule is predominantly in a fixed, mostly-extended orientation, other domain conformations are feasible but are not “preferred” without some chemical, biochemical, or mechanical signal (Marino et al., 2006; Pinotsis et al., 2006; Zou et al., 2006; Lee et al., 2007). This moderately stiff linker was unexpected, since generally titin has been imagined as a series of beads (Ig and FnIII domains) on a string (the linkers between the domains). Based on Z1Z2 and other structures (discussed below), such a model is clearly overly simplistic (Marino et al., 2005, 2006). Several studies now show that the orientation of Ig domains relative to their neighbors is not stochastic, but is instead usually fixed, and is dictated by the chemical make-up of both the linker region and the interdomain loops that comprise the domain/domain interface. (Marino et al., 2005; Mueller et al., 2007; Bucher et al., 2010).

One teleological possibility why this Z-region of titin would preferentially maintain a fixed, extended domain-domain orientation is to promote ligand binding and specificity. This hypothesis is suggested through a comparison of unbound-Z1Z2 structures to the structure of Z1Z2 bound to its molecular target telethonin. (Mues et al., 1998; Zou et al., 2006). This Z1Z2-telethonin complex helps to orient titin in the sarcomere, and is responsible for aligning titin in a direct head-to-head orientation at the Z-line. In comparing structural data of the unbound and bound versions of Z1Z2, it is obvious that unbound Z1Z2 adopts an extended conformation that is close to its ligand-bound conformation. The presence of a rigid, extended linker could therefore either assist in orienting Z1Z2 to participate in productive ligand binding, and/or assist in making ligand binding more energetically favorable.

The functional ramifications of the titin Z-disk interactions are a point of debate. In one theory, titin's high affinity to its Z-disk target is posited make this region a poor mechanosensor; it would require too much force to directly pull this protein-protein interaction apart (Gautel, 2011b). On the other hand, the titin-telethonin interaction is only mechanically strong in one direction (Bertz et al., 2009). The Z-disk morphology changes from “small square” to a “basketweave” upon muscle activation, and this change in macromolecular structure should result in other mechanical forces such as stress, shear stress, and torque being exerted on proteins within the Z-disk (Perz-Edwards and Reedy, 2011). Thus, while titin itself is normally thought of as a length sensor that responds to stretch, the Z-disk portion of titin, and the Z1Z2-telethonin complex in particular, may respond to these other Z-disk-induced mechanical stimuli and correspondingly act as a tension sensor (Knoll et al., 2002; Zou et al., 2006; Bertz et al., 2009). This supposition is bolstered by the recent finding that telethonin does not play a role in mechanical stability of the sarcomeric Z-disc and probably is involved in mechano-signaling (Markert et al., 2010; Knoll et al., 2011). One intriguing possibility is that CSRP3 (MLP) and titin both interact with telethonin in a jigsaw puzzle-like manner to form a multimodal mechanosensor (Lee et al., 2006). Due to its length and modular architecture, titin can likely mediate signaling from multiple different kinds of sources in parallel. Thus, titin-mediated downstream signals that arise from different kinds of mechanical cues (for instance, stretch at the A-band and sheer stress at the Z-disc) are not mutually exclusive and in fact could transmit both differing and/or overlapping information.

### Titin structure in the I-Band

**Figure 3:**
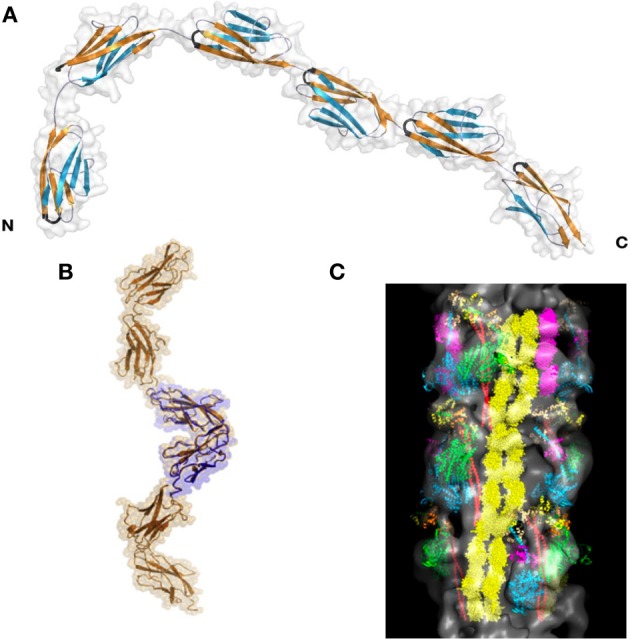
**Superstructure of titin. (A)** I65-I70, as determined via SAXS and X-ray crystallography. This structure shows both a “stiff,” extended segment, along with a hinge point. Reproduced with permission from Von Castelmur et al. (2008). **(B)** A SAXS model of the 11-domain super-repeat of titin in the A band. Here the helical twist of the titin molecule is clearly visible. Reproduced with permission from Tskhovrebova et al. (2010). **(C)** Shows this same titin super-repeat, in yellow, mapped onto a full thick filament, as it may appear *in vivo*. Reproduced with permission from Al-Khayat et al. (2013).

The titin I-band is comprised mostly of Ig domains (roughly 105 in total in this region), but also contains the PEVK region and the N2A and N2B region. Of these, the structures of many of the Ig regions have been solved, as has that of the PEVK region. Several papers show that various tandem Ig domains in the I band do not move freely relative to each other but, like in the Z-disc structures, are instead relatively fixed in an extended orientation (Improta et al., 1998; Marino et al., 2005). This physical feature is produced by the steric hindrance of the short linker between domains (2–3 residues) as well as domain-domain interactions at the Ig/Ig interfaces (Improta et al., 1998). Building on these data, Von Castelmur et al. published a rigorous study showing that a longer Ig filament (six linked Ig domains) also forms an elongated structure with occasional hinge points between specific Ig domains (Figure 3A) (Von Castelmur et al., 2008). From these data, the authors present a model where titin poly-Ig modules do not act like a chain or a string of beads, but behave more like a “carpenter's ruler” with discrete entropic points along the length of the filament. This same model may be more broadly applicable to other multi-domain modular proteins such as MyBP-C (Jeffries et al., 2011). Unlike the Z1Z2 domains, this extended I-band orientation does not engage in any known ligand binding. The straightening out of the hinge points at low forces (~5 pN), along with the extension of the PEVK region (discussed below) at slightly higher forces, is responsible for much of titin's elasticity at physiologically relevant stretching forces (Lee et al., 2007). There is some discussion as to whether this model can sufficiently explain all of titin's elastic properties (Politou et al., 1995), but no alternate theory that takes both structural data and the elastic property of the I-band into account has thus far been presented.

### PEVK

**Figure 4:**
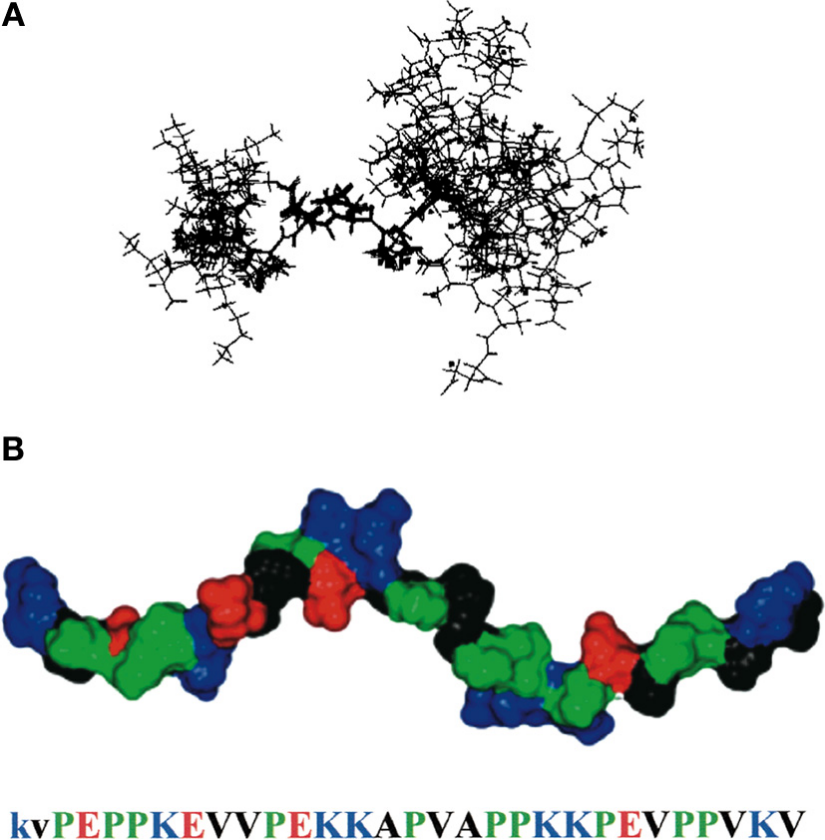
**PEVK structure. (A)** NMR ensemble of a 12-residue region of PEVK, showing both the polyproline type II helix and flanking unstructured regions. **(B)** model of the PEVK region, joining three short NMR structures together. Residues are colored according to their chemical properties (red is negative charge, blue is positive charge, green is proline, and black is hydrophobic). Reproduced with permission from Ma et al. (2001).

The PEVK region of titin, located in the I-band, is a 28-residue repeating sequence consisting mostly of P, E, V, or K and ranging in length from less than 200 residues in human cardiomyocytes to 2174 residues in human soleus muscle. Multiple proteins interact with this flexible region of titin, suggesting that the PEVK domain is a major link between biochemical signaling pathways and the physical stretching of the sarcomere. Among other interacting partners, PEVK can bind to PKG, associated with muscle relaxation, S100A1, associated with calcium release, and the nebulin SH3 domain (Politou et al., 1998; Yamasaki et al., 2001; Kruger and Linke, 2009). It was first hypothesized that the PEVK region of titin was uniformly disordered, and its purpose was solely to create the elastic resistance necessary for titin to elongate within the sarcomere. This model assumes a pure entropic spring mechanism to account for stretch resistance (Bang et al., 2001). Here, the polypeptide starts relaxed in a random orientation (a high entropy state). As the polypeptide chain is stretched, fewer conformations are allowed. This decreases the entropy and results in a higher total energy state and increased resistance (Nagy et al., 2005). This model can account for the soft elasticity of titin at low forces; however, Ma *et al*. published a series of papers showing that the PEVK region has partially structured elements interspersed amongst disordered regions (Figure 4).

At rest, each PEVK repeat contains short (6–10 residues) sequences of a moderately rigid polyproline type II helix, flanked by random coils (Ma et al., 2001). Cu^2+^ binds to specific regions in the PEVK repeat and alters the structure from a polyproline helix to β-turn (Ma and Wang, 2003a). Additionally, changes in environment, such as dielectric constant or temperature, transition the PEVK structure from a polyproline helix to a β turn or to a random coil (Ma et al., 2001; Ma and Wang, 2003a,b). Likewise, protein binding can significantly influence PEVK structure and therefore elasticity (Ma and Wang, 2002; Ma et al., 2006). None of these changes in PEVK structure are cooperative, either within a PEVK repeat or between PEVK repeats. Thus, in this revised model, each PEVK repeat contributes to the soft elasticity of titin at weak forces (5–50 pN) through a gradual conversion along the length of the PEVK segment from a helix or a β-turn to an elongated peptide. In essence, the PEVK region functions as a modified entropic spring, containing sections of quasi-stable structures that are tunable based on changes in intracellular conditions.

### N2A and N2B

Most titin isoforms contain a N2A and/or a N2B area that is N-terminally situated to the PEVK region. While the sequence and binding partners can vary between isoforms, these regions share an overall organization of 3–4 Ig domains interspersed amongst presumably disordered sequences. As discussed earlier, there are large macromolecular conformational changes in the I-band region of titin upon stretch and relaxation. The N2A/N2B/PEVK region, containing an abundance of partially unstructured and flexible sequences, is particularly sensitive to these changes. It is therefore not surprising that this area is involved in multiple non-sarcomeric signaling pathways that are influenced by stretch signaling. For instance, FHL-1 mediates Erk-2-regulated phosphorylation of an inter-Ig region of titin (Raskin et al., 2012). The FHL-1/titin interaction connects extracellular signals to transcription via stretch response (Sheikh et al., 2008). Other titin binding partners to N2A and N2B interact with the Ig domains instead of unstructured regions; for instance, P94/calpain 3 binds at Ig domains I82 and I83 to mediate sarcomeric remodeling (Sorimachi et al., 1995; Beckmann and Spencer, 2008). A more complete list of binding partners can be found in Kontrogianni-Konstantopoulos et al. (2009).

### Titin structure in the A-Band

The I-band and A-band segments of titin are mostly made up of modular Ig domains or of both FnIII and Ig domains, respectively. Despite this apparent similarity in structural organization, there is now good experimental evidence that these two regions of titin behave differently in solution. Tandem domains from the A-band have more extensive and more structured domain/domain interfaces than those from the I-band (Mueller et al., 2007; Bucher et al., 2010). As in the I-band, the domain/domain interfaces within the titin A-band region seem to dictate the overall solution conformation (Bucher et al., 2010). At the A-band/M-line interface, the domain linker becomes significantly more structured and assumes a β-sheet conformation (Mueller et al., 2007; Steward et al., 2012).

The A-band of titin contains multiple super-repeats of 7 or 11 FnIII and Ig domains. Low and high-resolution structural work on an 11-domain super-repeat suggests that this segment of titin adopts an extended helical orientation, and likely can form a homodimer (Figure 3B) (Mueller et al., 2007; Bucher et al., 2010; Tskhovrebova et al., 2010). These data agree well with the current understanding of how the thick filament is organized, and in fact this helical titin homodimer easily is incorporated into a detailed model of the complete thick filament (Figure 3C) (Al-Khayat et al., 2013). It is thus likely the A-band super-repeats of titin exist in roughly the same conformation when partnered with their natural binding partners as they do alone in solution.

### Titin structure in the M line

The M-line of the sarcomere contains the titin kinase domain, along with 10 titin Ig domains that are separated by unstructured M-insertions of varying lengths. Individual high-resolution structures of the Ser/Thr kinase domain, M1, M4, M5, M7, and M10 have been solved (Table 1). M10 has also been solved in the presence of a binding partner, the Ig1 domain of obscurin-like protein (OL1) (Pernigo et al., 2010; Sauer et al., 2010). There is virtually no difference in the M10 domain alone or bound to target (RMSD 0.66 Å), even at the ligand interface. The M10-OL1 interaction occurs on one of the long axes of the M10 molecule. The interaction is driven through hydrophobic interactions (TΔS of binding = 12.0 kcal/mol; ΔH = 3.87 kcal/mol) and the interface is stabilized through multiple backbone hydrogen bonds between the B strand of titin and the G strand of OL1 (Pernigo et al., 2010). Several features of this binding event, including the presence of inter-subunit H-bonds between β-strands, the stabilization of binding via hydrophobic moieties, and an Ig domain that shows only minimal structural and side-chain rearrangement upon binding, are also found in the only other high-resolution structure of titin bound to its target, the Z1Z2–telethonin structure (Marino et al., 2006; Zou et al., 2006; Pernigo et al., 2010; Sauer et al., 2010).

The Ser/Thr kinase domain of titin is categorized in the myosin light chain kinase family. While this region is still classified as a pseudo-kinase, there is emerging evidence that it functions in mechanochemical signal transduction. This region consists of a regulatory domain containing a putative phosphorylation site and a calmodulin binding region, and a catalytic domain that binds both ATP and substrate (Mayans et al., 1998; Grater et al., 2005). Kinase activation is hypothesized to require a mechanical force exerted on the domain. This mechanical strain physically opens up the ATP active site, which then allows the titin kinase domain to phosphorylate itself and possibly also downstream effectors (Lange et al., 2005; Puchner et al., 2008). This mechanism is thus far only supported by *in vitro* data. Yet, while still unproven via knock-in or knock-out models, it is one of the most clearly articulated examples of how mechanical stimuli could be translated into chemical signals using a single protein. Kinase domain mutations cause hereditary myopathy with early respiratory failure (HMERF) (Lange et al., 2005). Recent evidence of mutations outside of the kinase domain can also cause this same disease. These findings suggest that the molecular mechanism of kinase stretch activation is more complicated than first suspected, and may involve multiple other sites within titin (Ohlsson et al., 2012; Pfeffer et al., 2012). A more extensive review of the kinase domain can be found in Kontrogianni-Konstantopoulos et al. (2009); Gautel (2011a); Temmerman et al. (2013).

## Obscurin

Obscurin can bind to both titin and small ankyrin, and is thus the only protein that links the sarcomeric cytoskeleton to the surrounding sarcoplasmic reticulum membrane system (Figure 2). Like titin, obscurin is comprised of a series of modular Ig and FnIII domains. The extreme C terminus of obscurin isoform A is nonmodular and is involved in binding small ankyrin (Young et al., 2001; Kontrogianni-Konstantopoulos et al., 2003; Kontrogianni-Konstantopoulos and Bloch, 2005; Borzok et al., 2007). Obscurin localizes to both the Z-disc and the M-line in myofibrogenesis, and primarily at the M-line in adult myocytes (Bang et al., 2001; Young et al., 2001; Kontrogianni-Konstantopoulos et al., 2003). However, this pattern is not universally agreed upon, in part because antibodies against different obscurin epitopes give conflicting information regarding localization (Kontrogianni-Konstantopoulos and Bloch, 2005; Kontrogianni-Konstantopoulos et al., 2009). These contradictory findings raise questions about the importance and frequency of splice variants (Bowman et al., 2007; Kontrogianni-Konstantopoulos et al., 2009). When antibodies against both the N and C-terminus are used simultaneously, obscurin stains in a reticular pattern that suggests it is positioned on the surface of the myofibril rather than impregnated through it (Kontrogianni-Konstantopoulos et al., 2003).

### Obscurin Ig domains

Eleven Ig domains of obscurin A have been solved individually, mainly through NMR analysis (Table 1). In addition, six Ig domains that belong to a splice variant have been solved. All of these domains are highly similar, with average pairwise RMSD of 1.4 Å. While the linker sequence between I-band domains of titin domains usually ranges between 2–5 residues, obscurin has no obvious linker sequence (Kontrogianni-Konstantopoulos et al., 2009). At least one obscurin binding partner, the ZIg9/10 domain of titin, requires tandem obscurin Ig domains for interaction (Young et al., 2001). Thus it is likely that, like titin, relative orientation between obscurin domains can be important for target binding.

Obscurin creates the only known link between the sarcomplasmic reticulum and the contractile apparatus. This unique positioning points to a role for obscurin in both membrane and cytoskeletal organization; obscurin knockout and knockdown experiments show dysregulation to both the sarcolemma and to lateral sarcomeric organization (Raeker et al., 2006; Raeker and Russell, 2011; Randazzo et al., 2013). The (presumably) semi-flexible tandem Ig domains that comprise most of obscurin also present an obvious mechanism to protect the myofibril against repeated contractions and stretches. In one such model, obscurin can transduce the force of muscle contraction onto the surrounding membrane and cytoskeletal system while simultaneously dampening this potentially destructive mechanical force via modulation of the Ig-Ig architecture.

### Other obscurin domains

Obscurin contains several non-Ig or FnIII-like domains near the C-terminus of the molecule, and of these, the Pleckstrin Homology domain (PH) and an SH3 domain of obscurin have high resolution structures associated with them (Blomberg et al., 2000). The SH3 domain is typical in structure, with no currently known binding partners. Neighboring this domain is a RhoGEF module and the PH domain (Blomberg et al., 2000). These tandem domains can bind to Ran binding protein 9 and RhoA, and are involved in GTPase signaling, actin remodeling and actin-membrane linkages, and modulation of protein synthesis (Bowman et al., 2008; Ford-Speelman et al., 2009). RhoA can be activated via multiple upstream events, including changes in ion concentrations and other signals. Thus obscurin activation of RhoA is the first link between intracellular sarcomeric function and other extracellular factors, all of which can apparently share at least some of the same cellular response pathways (Ford-Speelman et al., 2009) [reviewed in Miyamoto et al. (2010)].

Nearby in sequence to the RhoGEF and PH domains, an IQ domain suggests a link between obscurin and calmodulin signaling. At the C-terminus of the molecule some obscurin isoforms have two kinase domains, for which there are currently no structures. In *c. elegans* these domains can bind to both LIM-9 and SCPL-1. This is an alternative link between the membrane network and the contractile apparatus (Qadota et al., 2008; Xiong et al., 2009; Warner et al., 2013). The full function of the obscurin kinase domains is still unclear, although based on sequence similarity domain 1 may be similar to MLCK and domain 2 is probably a pseudo kinase. Structure prediction programs suggest that these domains may be more similar to titin/twitchin kinase domains, and thus may have a mechanosensing role. The presence of the SH3, PH, RhoGEF, and kinase domains reveal obscurin to be a focal point in sarcomeric signaling; however the precise targets, and therefore the exact nature of these signals has yet to be described. For more information on obscurin function and role in myocytes, please see (Kontrogianni-Konstantopoulos et al., 2009; Gautel, 2011b).

## Nebulin

**Figure 5:**
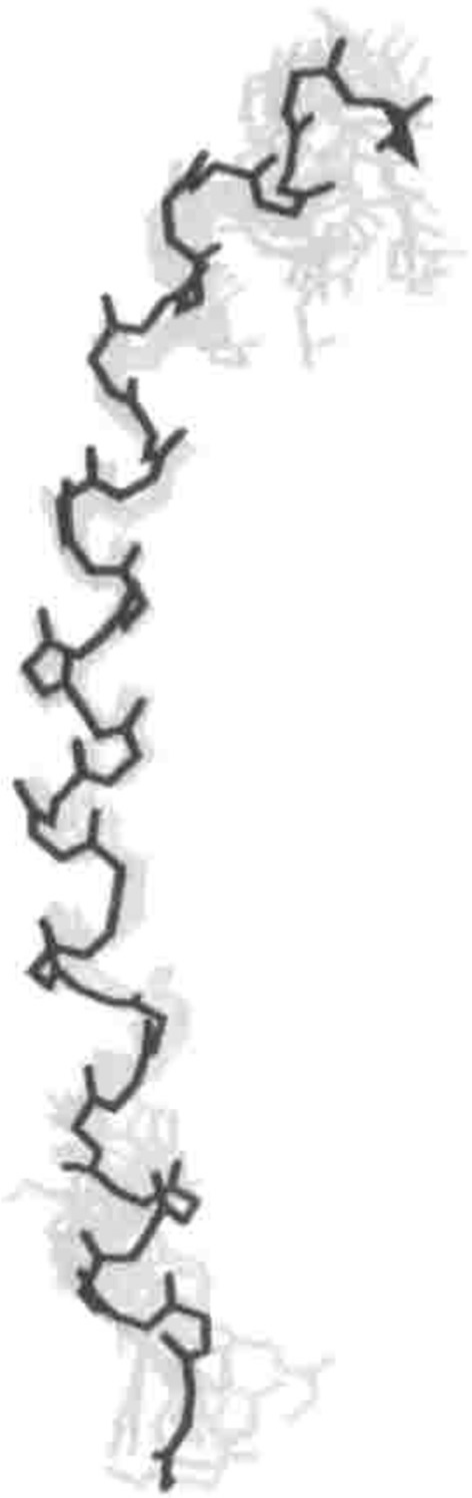
**NMR solution structure of one representative complete nebulin repeat (37 residues)**. The RMSD of this structure is 0.9 Å. Reproduced with permission from Pfuhl et al. (1994).

Nebulin (500–800 kDa) is comprised almost entirely of a simple ~35 residue repeat that tightly associates with the thin filament via the central consensus sequence SDxxYK (Jin and Wang, 1991a,b). Almost 20 years ago, CD and NMR studies showed that the entirety of the nebulin repeat assumes an α-helical conformation (Figure 5) (Pfuhl et al., 1994). Such a structure allows each nebulin repeat to extend ~5.5 nm, which is also the length of the actin monomer within the thin filament (Labeit and Kolmerer, 1995; Suzuki et al., 2000). There are two nebulin molecules associated with each actin polymer, one for each face of the filament. The number of actin subunits in the thin filament and the number of nebulin repeats (in each case, at least 150 per thin filament), combined with the high affinity (nM range) between nebulin and actin suggest a nebulin-actin avidity that is unlikely to ever dissociate *in vivo* (Jin and Wang, 1991b; Labeit et al., 1991; Pfuhl et al., 1996; Wang et al., 1996). Further sequence analysis shows that nebulin repeats can be grouped into 22 sections of seven repeats, with each “super repeat” predicted to be the length of a single tropomyosin molecule associated with seven F-actin monomers, ~38.5 nm (Korn, 1982; Kruger et al., 1991; Wang et al., 1996). This coincidence of lengths, plus the fact that nebulin also binds to tropomyosin and other actin-associated proteins, furthers the idea that nebulin is fully integrated into the structure of the thin filament (Jin and Wang, 1991a,b; Root and Wang, 1994; Wang et al., 1996). Such an association is likely crucial for two of nebulin's functions: stabilizing actin polymers and dictating thin filament length (Kruger et al., 1991; Labeit et al., 1991; Bang et al., 2006; Witt et al., 2006). Due to its close association with F-actin and lack of enzymatic activity, nebulin is the only giant muscle protein that is not directly involved in muscle elasticity.

Nebulin is oriented in the sarcomere so that the N-terminus is in the I-band, closely associated with the thin filament. The C-terminus is embedded into the Z-disc. This configuration both regulates the Z-disc structure and anchors nebulin to the surrounding cytoskeleton (Millevoi et al., 1998). The C-terminal ~18 nebulin repeats are at least partially embedded in the Z-disc, and are not part of a super-repeat motif. In this region, the SDxxYK sequence is not as conserved as in the rest of the molecule, and consequently this region does not bind to actin. A much shorter isoform, nebulette, is present in cardiac cells at the Z-disc (Millevoi et al., 1998). The 100 kDa nebulette protein is similar to nebulin at the C-terminus, including the presence of an SH3 domain (Eulitz et al., 2013). However, nebulette only has 23 copies of the characteristic nebulin repeat, and is only predicted to extend 150 nm away from the edge of the Z-disc (Millevoi et al., 1998; Littlefield and Fowler, 2008). Like nebulin, nebulette likely acts by stabilizing the thin filament, and plays a role in Z-disk organization (Littlefield and Fowler, 2008; Pappas et al., 2011).

Structural homology predictions suggest that these nebulin repeats are helical, and these repeats can bind to other Z-disc structural proteins such as α-actinin, CapZ, archvillin, and desmin (Nave et al., 1990; Bang et al., 2002; Witt et al., 2006; Lee et al., 2008). C-terminal of these repeats is a serine-rich domain that is a potential phosphorylation site of nebulin, followed by an Lasp-like SH3 domain that binds proline-rich sequences in CapZ, myopalladin, and the PEVK region in titin (Politou et al., 1998, 2002; Bang et al., 2001; Pappas et al., 2008). When taken as a whole, nebulin's structural motifs describe a well-anchored, long molecule that has evolved to stabilize and organize a large number of structural features of the thin filament.

## Conclusions

There have been two major breakthroughs in elucidating giant muscle protein structures. First was the realization that individual domains can be studied outside the context of the entire molecule. This allowed for study of multiple X-ray and solution structures of various giant muscle protein segments. Next was the ability to combine high-resolution structures with other structure/dynamics experiments. From these analyses, we now have a more nuanced insight into how the position of one domain relative to its neighbor dictates overall protein shape, flexibility, and target binding. Current questions involve the structure/function/dynamics relationship of giant muscle proteins. For instance, what factors drive target protein binding to one domain but not the neighboring domain? What dictates the properties of the “hinge regions” of titin, and where are these hinges located? Does obscurin also behave like a carpenter's ruler? How does obscurin affect the movement and stretch of muscle? Additionally, many of the enzymatic functions of titin and obscurin are only now beginning to come to light. Elucidating the full molecular mechanism of the titin kinase domain is of key importance for understanding how titin integrates with the rest of the cellular machinery. Likewise, determining the binding partners of obscurin's SH3 domain, fully understanding the downstream effectors of the RhoGEF/PH domain, and studying further the potential mechanosensing roles of the obscurin kinase domains are all areas of interest for understanding the enzymatic roles of giant muscle proteins. Finally, nebulin's role in Z-disc organization and mechanosensing is only now being studied. Answers to these unresolved items will involve a combination of structure and functional studies. The process of more fully understanding such relationships will undoubtedly lead to a more complete view of how these molecular giants intertwine with the rest of the sarcomeric machinery.

### Conflict of interest statement

The authors declare that the research was conducted in the absence of any commercial or financial relationships that could be construed as a potential conflict of interest.
